# Nicotinamide Riboside Enhances Mitochondrial Proteostasis and Adult Neurogenesis through Activation of Mitochondrial Unfolded Protein Response Signaling in the Brain of ALS SOD1^G93A^ Mice

**DOI:** 10.7150/ijbs.38487

**Published:** 2020-01-01

**Authors:** Qi Zhou, Lei Zhu, Weiwen Qiu, Yue Liu, Fang Yang, Wenzhi Chen, Renshi Xu

**Affiliations:** 1 Department of Neurology, Jiangxi Provincial People's Hospital, Affiliated People's Hospital of Nanchang University, Nanchang 330006, Jiangxi, China; 2 Department of Neurology, First Affiliated Hospital of Nanchang University, Nanchang 330006, Jiangxi, China

**Keywords:** Nicotinamide riboside, Amyotrophic lateral sclerosis, Mitochondrial unfolded protein response related protein, Neural stem cells, Neuronal precursor cells

## Abstract

Amyotrophic lateral sclerosis (ALS) is caused by the progressive degeneration of motor neurons in the spinal cord, the brain stem, and the motor cortex. So far, there is still a lack of effective drugs. Nicotinamide adenine dinucleotide (NAD+) takes part in redox reactions and the NAD-dependent signaling pathway. The NAD+ decline is related with many neurological diseases, leading to the accumulation of neurotoxic protein in the central nervous system. Moreover, the NAD+ supplementation is shown to promote neural stem cells/neuronal precursor cells (NSCs/NPCs) pool maintenance. Regulatory mechanisms and functions of NAD+ metabolism in ALS are still unknown. Thus, we hypothesized the aggregation of human SOD1 toxic protein and the fate of NSCs/NPCs in the ALS disease could be improved by the administration of nicotinamide riboside (NR), an NAD+ precursor. In this study, we treated SOD1^G93A^ transgenic and wild-type mice by the oral administration of 20 mg/ml NR starting at 50 days of age. Effects of NR on the body weight, the motor function, the onset and the survival were assessed during the experiment. The expression of mutant hSOD1 protein, mitochondrial unfolded protein response (UPR^mt^) related protein, mitophagy markers and NAD+ metabolism related protein were detected by immunoblotting. Effects of NR on the NSCs/NPCs in neurogenic niches of brain were identified by the immunofluorescence staining. Our investigation elucidated that the NR treatment exhibited better hanging wire endurance but did not postpone the onset or extend the life span of SOD1^G93A^ mice. Besides, we observed that the NR repletion promoted the clearance of mitochondrial hSOD1 neurotoxic protein. Meanwhile, the mitochondrial function pathway was disrupted in the brain of SOD1^G93A^ mice. What's more, we demonstrated that the inadequate function of NAD+ salvage synthesis pathway was the primary explanation behind the decline of NAD+, and the NR treatment enhanced the proliferation and migration of NSCs/NPCs in the brain of SOD1^G93A^ mice. At last, we found that levels of UPR^mt^ related protein were significantly increased in the brain of SOD1^G93A^ mice after the NR treatment. In summary, these findings reveal that the administration of NR activates UPR^mt^ signaling, modulates mitochondrial proteostasis and improves the adult neurogenesis in the brain of SOD1^G93A^ mice.

## Introduction

NAD+, known as active metabolite types of vitamin B3, is a fundamental little molecule co-factor in metabolic redox responses 1, conveying high vitality electrons to help oxidative phosphorylation by reversibly oxidizing or lessening NAD+ 2, 3, and filling in as a substrate for NAD-subordinate compounds that connect cell metabolism with the epigenetic guideline and the DNA damage repair 3. Mammalian cells make NAD+ by three distinct methods: (1) De novo synthesis from the tryptophan; (2) Generation from the nicotinic acid using the Preiss-Handler (PH) pathway; or (3) Synthesis from nicotinamide (NAM) or NR via the salvage pathway 1, 4.

The NAD+ biosynthesis mediated by the nicotinamide monophosphoribosyl transferase (NAMPT) and the NAD+ utilization by NAD+-consuming enzymes are in a sensitive balance 5. The decrease of NAD+ indicates the dysfunction of basic physiological system of whole body 6. The ongoing development of understanding that the NAD+ homeostasis is vulnerable to aging and disease processes 7, 8, has invigorated tests to determine whether the replenishment of cellular or tissue NAD+ improves disease phenotypes in neurodegeneration-related diseases.

Mitochondria, the primary site of cellular energy acquisition, are gotten from proteobacteria that developed within our cells in endosymbiosis. The significant capacity of mitochondria is the creation of adenosine triphosphate (ATP) through the oxidative phosphorylation system (OXPHOS) 9. During the cell respiration, electrons are exchanged to oxygen atoms and produce superoxide anions. Since they are exceptionally poisonous, superoxide anions are generally neutralized by antioxidant enzymes. However, the mitochondrial dysfunction prompts to the ATP depletion, the superoxide anion over-burden and release of proapoptotic molecules, such as cytochrome c in pathological conditions 9. Cells have nevertheless adapted a mitochondrial quality control (MQC) framework, which including the mitochondrial biogenesis, mitochondrial derived vesicles, mitochondrial dynamics, mitophagy and UPR^mt^ in the mammalian, to defeat mitochondrial defects 10, 11. Thus, MQC is especially crucial for neurons which are long living cells and relatively easily lead to the accumulate damage in mitochondria in a state of stress 12.

The loss of mitochondrial proteostasis has been proposed to assume an important role in the age-related decline 13, 14. Recent studies have implicated a mitochondrial stress reaction, the UPRm, as a connection between mitochondrial proteostasis and aging in different organisms 15, 16. The UPR^mt^ is a vigorous transcriptional reaction that has been proposed to alleviate the proteostatic stress in mitochondria by promoting folding, restricting import, and diminishing the translation of mitochondrial proteins 17. In addition, a related basic pathway of mitochondrial damage in many situations is the consumption of NAD+, which occur by the initiation of pathways that utilize up cellular and mitochondrial NAD+ pools, such as the actuation of poly (ADP-ribose) polymerases (PARPs) and the cyclic ADP-ribose hydrolase (CD38) 18, 19. And it replenishes the NAD+ pool to counteract carbon stress by improving the activity of sirtuins, or forestalling the oxidative damage with antioxidants, helping to keep up the mitochondria proteostasis 20.

Several lines of proof support the role of NAD+ supplementation for reestablishing function of NSCs/NPCs during ageing and with regards to neurodegenerative diseases. NAD+ levels decrease with age in the hippocampus of mice, alongside the expression of NAMPT, an enzyme associated with the NAD+ salvage pathway 21. The deletion of NAMPT in NSCs diminishes the proliferation and self-renewal ability in the adult hippocampus, which could be protected with the nicotinamide mononucleotide (NMN) supplementation 21. The NAD+ repletion with the NR supplementation advances the NSC proliferation and salvages neurogenesis in the ageing subventricular zone (SVZ) and the hippocampal dentate gyrus (SGZ) 20. The NR supplementation in a 3xTg-AD mouse that contains the insufficient POLG displayed the less phosphorylated tau pathology, diminished the DNA damage, expanded the survival of hippocampal neurons and improved the cognitive function 22. All above proofs shows that NAD+ metabolic homeostasis are central regulators of NSCs/NPCs destiny decision, which stimulate us to interest in potential advantages enhancing the tissue NAD+ as a way to treat neurodegenerative diseases.

ALS is caused by the progressive degeneration of motor neurons in the spinal cord, the brain stem and the motor cortex. The motor neuron death prompts the muscle weakness and paralysis, causing death in 1-5 years from the time of symptom onset. Most ALS cases are sporadic ALS (SALS), and the introduction to yet unidentified natural toxicants might be responsible for SALS 23. About 5-10% of cases are the inherited familial ALS (FALS), and the primary identified ALS-linked gene was the superoxide dismutase 1 (SOD1) 24. Mutations in the SOD1 gene represent up to 20% of FALS and 1-2% of obviously SALS cases. Mutations in several other genes have now been recognized in many FALS pedigrees 23, 25, 26. Each mutated gene has its own genetic and molecular signature, FALS and SALS are phenotypically unclear yet, and a noteworthy share of our understanding originates from the investigation of rodents overexpressing the ALS-linked mutant SOD1 that develops an ALS-like phenotype 27. Various molecular pathways have been involved in the neuronal death in ALS. Only two disease-modifying therapies currently approved by US Food and Drug Administration (FDA) for ALS are riluzole and edaravone, but the exact neuroprotective action of drugs is obscure and has no obvious improvement on the quality of life 28-30. The absence of alternative drugs for the treatment of ALS requests the improvement of new remedial methodologies. Recently, it is exhibited the SOD1 protein misplacing and mitochondrial dysfunction in the brain of ALS mice 31. Besides, several lines of evidences suggest the NSCs/NPCs niche is modified in ALS mice 32, 33. Notably, the enhancing NAD+ salvage pathway returns the lethality of primary astrocytes expressing ALS-linked mutant superoxide dismutase 1 34. In line with this observation, NAD+ salvage pathway proteins suppress the proteotoxicity in yeast models of neurodegeneration by advancing the clearance of misfolded proteins 35. On the contrary, the deletion of NAMPT in projection neurons coming about of NAD+ decrease of adult mice prompts the motor dysfunction, neurodegeneration, and death 36. Collectively, these discoveries strongly suggest that the improving NAD+ biosynthesis by administering NAD+ precursors, might be a candidate therapeutic target against the ALS disease related to the mitochondrial dysfunction and the adult neurogenesis.

In this study, our investigation elucidated the NR treatment exhibited better hanging wire endurance but did not postpone the onset or extend the life span of SOD1^G93A^ mice. Besides, we demonstrated that the replenishment of intracellular NAD+ by providing NR could reduce neurotoxic protein aggregates of mitochondria in the brain of SOD1^G93A^ mice, and the mitochondrial function pathway was disrupted in the brain of SOD1^G93A^ mice. Meanwhile, we suggested that the NAMPT-mediated NAD+ biosynthesis was the main reason for the NAD+ levels decline and the de novo biosynthesis of NAD+ might act as an adaptive response of body in the ALS. As we predicted, we demonstrated that the NR treatment enhanced the proliferation and migration of NPCs/NSCs in the brain of SOD1^G93A^ mice. What's more, we found that levels of UPR^mt^ related protein were significantly increased in the brain of SOD1^G93A^ mice after the NR treatment. At last, we concluded that the NR might modulate the mitochondrial proteostasis and improve the adult neurogenesis through activating the mitochondrial UPR signaling in the brain of SOD1^G93A^ mice.

## Materials and methods

### Animals, treatments and sample

Transgenic mice of SOD1 (G93A) mutation on a C57BL/6 foundation (Jackson laboratory, Bar Harbour, Maine) that harbor the high copy number of mutant allele hSOD1 were kept up as a hemizygous line in an SPF-reproducing facility (The neurological lab of first affiliated hospital of Nanchang university). The hemizygous line was kept up by mating transgenic males with C57BL/6 WT females. All creatures were housed in a room with controlled photoperiod (08:00-20:00 light) and temperature (22±1ºC) with free access to the standard nourishment and water. Wild-type (WT) and transgenic (TG) mice were recognized by numbering toe marks. Preceding the begin of different experiments, they were randomly apportioned to different treatment groups.

NR was specially synthesized as the previously described 37, and purified by the reverse-phase HPLC. The mother liquor of NR was obtained by adding 10 ml 0.9% saline to 6 g NR powder with purity > 98%. The concentration of NR mother liquor was 600 mg/ml. After the NR powder completely dissolved, 290 ml distilled water was added to the 10 ml mother liquor to set up a 20 mg/ml NR working fluid. For the drug treatment, NR was administered to mice from postnatal 50 days through drinking water, provided in light-protected bottles. Based on drinking water measurement taken in hSOD1^G93A^ mice, NR dosages for every one equated to 400 mg/kg/day.

For animal grouping, mice were randomly divided into 4 groups: (1) SOD1-G93A TG mice receiving NR treatment (G93A-NR); (2) Litter-matched SOD1-G93A TG mice receiving NR-free drinking water (G93A-Vehicle); (3) WT control mice receiving NR treatment (WT-NR); (4) Litter-matched WT mice receiving NR-free drinking water (WT-Vehicle). For the immunohistochemistry analysis, n=3 mice were for each group. For the western blot analysis, n=3 mice were for each group. For the animal behavior test, n=4 mice were for each group, and the drug treatment was continued daily until the end-stage of disease (i.e. point of euthanasia). To evade any potential bias, all drug and vehicle treatments were coded and afterward administered and subsequently investigated by a researcher blinded to treatment groups. Decoding only happened after each animal experiment was finished. All animal studies were performed in accordance with the Guide for the Care and Use of Laboratory Animals of China. All experiments involving mice were checked and affirmed by the ethics committee for animal care and utilization of the first affiliated hospital of Nanchang University, China.

### Behavioral recording

Every three days, mice underwent the hanging wire test and the weight measure. Mice were trained one week before treated, and data were gathered from 70 days of age. For the hanging wire test, the latency of mice to fall from a wire cage top, which was gradually inverted and suspended at roughly 30 cm to the floor, was utilized as a record of motor weakness. The test was repeated three times to obtain the mean estimation of three trials. The disease onset was characterized by age at which hind limb tremors were apparent while suspending the mouse in the air by its tail. The survival was determined by the failure of mice to right itself inside 15-30 seconds if laid on either side. This is a broadly accepted end point for life expectancy studies in ALS mice 38, 39, and ensures that mice are killed before they are unfit to reach food or water. Mice were executed by the cervical disengagement following anesthesia.

### Immunofluorescent staining

Mice were anesthetized and perfused utilizing 20 ml of 0.9% saline and 40 ml of 4% paraformaldehyde in 1xPBS (pH 7.5) at the room temperature. Brain was extracted and set in 4% paraformaldehyde buffer overnight, hatched in 20% sucrose in 1xPBS (pH 7.5), embedded utilizing the optimum cutting temperature (OCT). Slices were coronally and progressively cut into 12 μm sections from the rostral to the caudal on a leica cryostat and gathered on superfrost plus slides. In the fluorescent immunohistochemical stain, areas of SVZ, SGZ and olfactory bulb (OB) tissues were permeabilized utilizing 0.2% TritonX-100 and blocked utilizing 10% goat serum in 1xPBS after rehydrated in 1xPBS (pH7.4), incubated utilizing primary antibodies: Vimentin 1:100, Doublecortin (DCX) 1:200 (Abcam, Hong Kong Ltd.) at 4°C overnight, trailed by washing 6 times with 0.2% Triton X-100 in 1xPBS, incubating utilizing secondary antibodies (Donkey anti rabbit, 1:200) conjugated to the fluorescence rhodamine (Red) for 2 hours at the room temperature, and the DAPI recolor (Blue), widely washing for 5 times, each for 5 minutes, mounting utilizing the antifade medium, examining in a Nikon E800 fluorescent magnifying instrument outfitted with a spot digital camera (Diagnostic Instruments, Sterling Heights, MI, USA) and Photoshop software (Adobe Systems, San Jose, CA, USA), and taking pictures. Multiple labeled histochemical stains conjugated to Vimentin, DCX and DAPI were utilized to watch and analyze the cell proliferation and migration. The analysis of immunohistochemical positive cells was performed by counting amount of positive cells in sections of SGZ, SVZ and OB at 200 magnifications in 3 sections and computing positive cells sum of all 3 sections, then the sum was divided by the section number, 3 mice per group were utilized, the averaged amount was utilized for the quantitative investigation.

### Protein preparation

#### Total protein fractions

For the absolute protein extraction, brain tissues acquired from WT-Vehicle vs. WT-NR or G93A-Vehicle vs. G93A-NR mice were washed in PBS (0.1 mmol/L), and homogenized with the buffer [Tris-HCl pH 7.5, 20 mmol/L; NP-40, 1%; EDTA, 2 mmol/L; PMSF, 2 mmol/L; Triton-100, 1%; aprotinin, 25 g/ml; pesptatin A, 10 ug/ml; leupeptin, 50 μg/ml; and dithiothreitol (DTT), 2 mmol/L] with the protein extraction reagent enhanced with a protease inhibitor cocktail (Animal tissue whole protein extraction kit, Solarbio, Beijing, China). Homogenates (Total fractions) were then solidified/thawed three times in the fluid nitrogen and centrifuged at 20,800×g for 10 minutes, at 4°C, in order to remove cell debris, the subsequent supernatant was gathered and stored at -80 °C for the later use.

#### Mitochondrial-enriched fractions

Fresh brain tissues acquired from WT-Vehicle vs. WT-NR or G93A-Vehicle vs. G93A-NR mice were washed with PBS, and the blood was washed out. The filter paper was sucked dry. Fragments were cut by scissors and put into a little volume glass homogenizer. Adding 1.0 ml ice pre-cooled lysis buffer (Mitochondrial extraction kit, Solarbio, Beijing, China). After homogenization, were centrifuged at 1,000×g for 12 minutes for three times (4°C) to pellet the nuclei and the cell flotsam and jetsam. The supernatant was additionally centrifuged at 12,000×g for 10 minutes (4°C) and the subsequent pellet (Mitochondrial-enriched fraction) resuspended in the supplemented 100 ml store buffer and stored at -80°C for the later use.

### Western blot analysis

Subsequent to mixing with the loading buffer and boiling at 100°C for 5 minutes, equivalent amounts of proteins were isolated by 8-12% sodium dodecyl sulfate-polyacrylamide gel electrophoresis (SDS-PAGE). Polyvinylidene fluoride (PVDF) membranes were utilized for the protein transfer and afterward incubated with relating primary antibodies overnight at 4°C after obstructing by 5% bovine serum or skim milk. Primary rabbit-derived antibodies included SOD1 (1:500, Proteintech, 10269-1-AP), LC3 (1:1000, Proteintech, 14600-1-AP), P62 (1:1000, Proteintech, 18420-1-AP), CLPP (1:3000, Proteintech, 15698-1-AP), HSP60 (1:1000, Proteintech, 15282-1-AP), LONP1 (1:3000, Proteintech, 15440-1-AP), NAMPT (1:5000, Proteintech, 11776-1-AP), NMNAT3 (1:1000, Proteintech, 13236-1-AP), and β-actin (1:10000, Proteintech, 20536-1-AP). After washing with the Tris-buffered saline-Tween (TBST) for 3 times (10 minutes each time), anti-rabbit HRP-linked secondary antibodies were added. Membranes were incubated at the room temperature for 1 hour and after washed with TBST for 3 times (10 minutes each time). The chemical luminescence was evaluated using the BeyoECL Plus Kit (Beyotime, Shanghai, China).

### Statistical analysis

Data were analyzed with the GraphPad Prism software (GraphPad Software, San Diego, CA). The Kaplan-Meier survival curve was analyzed using a Log-rank (Mantel-Cox) test. The comparison between two groups was performed by the Student's t test. All other data were analyzed with two-way ANOVA, followed by the Tukey's multiple comparisons post hoc test when appropriate. Data are expressed as mean + SEM as indicated. P-values below 0.05 were considered as significantly.

## Results

### The mutant hSOD1 protein incorrectly aggregated in the mitochondria of ALS SOD1^G93A^ mice brain tissue

**Figure 1:**
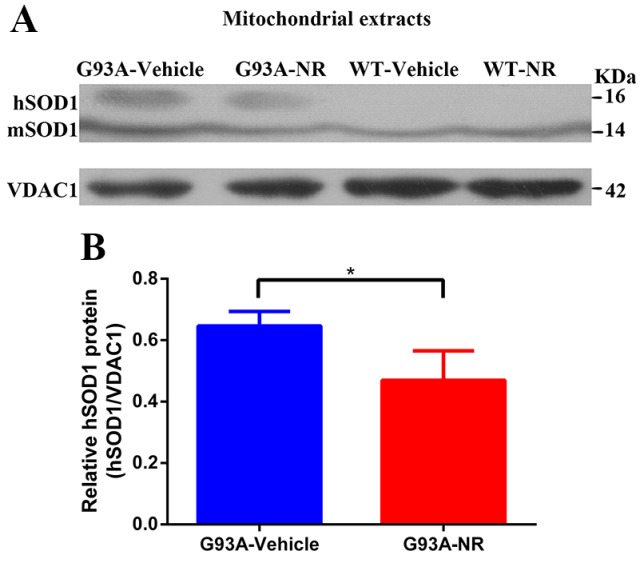
Mutant hSOD1 protein incorrectly aggregated in the mitochondria, and NR repletion promoted the clearance of mutant hSOD1 neurotoxic protein in ALS SOD1^G93A^ mice. **(A)** Representative western blots showed hSOD1 levels of mitochondria enriched fractions in four groups mice (G93A-Vehicle, G93A-NR, WT-Vehicle, WT-NR). **(B)** Bar graphs showed the quantification of relative protein density of hSOD1 in the mitochondria fraction. VDAC1 was shown as the loading control. Data were expressed as mean ± SEM of n=3 mice/group; *P<0.05.

As previously shown, the WT SOD1 protein is ordinarily restricted in both the cytoplasm and the nucleus of human cells 40, 41. And the mutant hSOD1 has been shown to associate with the mitochondria 42-46, exclusively in tissues from the central nervous system (CNS). To characterize the distribution of hSOD1 protein in the G93A-Vehicle and WT-Vehicle mice at the age of 120 days, hSOD1 protein levels of mitochondria-enriched portions were measured by the western blot analysis using mice brain tissues. As expected, our data demonstrated that there was obvious hSOD1 protein expression in the mitochondria of G93A-Vehicle group; however, we observed no mitochondrial hSOD1 protein expression in the WT-Vehicle group (Fig. 1A).

### The mitochondrial proteostasis was disturbed in the brain of ALS SOD1^G93A^ mice

**Figure 2:**
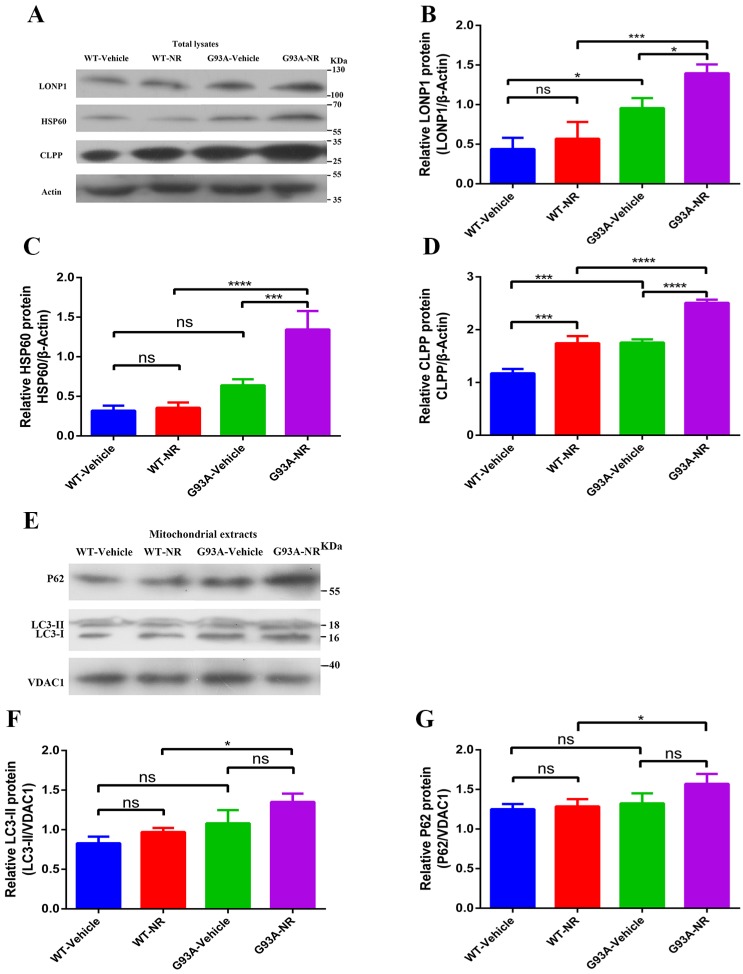
Mitochondrial proteostasis were disturbed in the brain of ALS SOD1^G93A^ mice. Representative immunoblots showed the expression of ClPP, HSP60, LONP1 (UPR^mt^ pathways) in total lysates fraction **(A)** and P62, LC3-II (Mitophagy pathways) in the mitochondria fraction **(E)**. Bar graphs showed the quantification of relative protein density of CLPP **(B)**, HSP60 **(C)**, LONP1 **(D)** in total lysates. Bar graphs showed the quantification of relative protein density of LC3-II **(F)** and P62 **(G)** in the mitochondria fraction. The protein density of CLPP, HSP60, LONP1 proteins was normalized with β-actin in total lysates. The protein density of LC3-II, P62 was normalized with VDAC1 in the mitochondria fraction. Data was showed as mean ± SEM of n=3 mice/group; *P<0.05, **P<0.01, ***P<0.001****P<0.0001.

Mitochondrial anomalies in ALS incorporate to decrease the mitochondrial respiration activity and change in the mitochondrial morphology 47, 48. However, the relevance of other aspects of mitochondrial homeostasis, such as the mitochondrial proteostasis, to the pathogenesis of ALS is still mostly unknown. Because the UPR^mt^ and mitophagy, two major MQC pathways, are connected with the mitochondrial proteostasis, we analyzed cortex samples of G93A-Vehicle and WT-Vehicle mice of ALS at the age of 120 days. The immunoblotting of total lysates from the brain cortex of G93A-Vehicle group showed significantly up-regulated expression of mitochondrial LONP1, HSP60 and ClPP protein, the representative of UPR^mt^ markers in vivo, relative to WT-Vehicle mice (Fig. 2B, C, D). However, we observed no statistical changes in the mitochondrial protein expression level of LC3-II, P62, the representative of mitophagy markers in vivo, between two groups (Fig. 2F, G).

### The inadequate NAMPT-mediated NAD+ salvage synthesis was the main explanation behind the decline of NAD+ in the ALS SOD1^G93A^ mice

**Figure 3:**
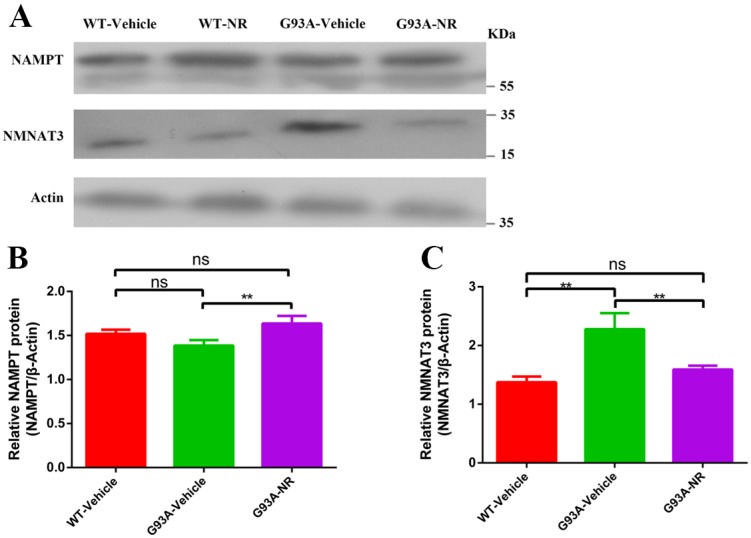
Inadequate NAMPT-mediated NAD+ salvage synthesis was the main reason for the decline of NAD+ in ALS SOD1^G93A^ mice.** (A)** Representative western blots showed NAMPT and NMNAT3 levels in three groups. Bar graphs showed the quantification of relative protein levels of **(B)** NAMPT, **(C)** NMNAT3 in total lysates. Protein levels of NAMPT and NMNAT3 protein were normalized with β-actin. Data were showed as mean ± SEM of n=3 mice/group; (*P<0.05, **P<0.01, ***P<0.001, ****P<0.0001).

To investigate the reason for NAD+ decline in the development and progression of ALS, we firstly evaluated protein expression levels related to the NAD+ homeostasis in G93A-Vehicle and litter-matched WT-Vehicle mice at the age of 120 days. Protein levels of key enzymes NAMPT (Representative of key rate-limiting enzymes of salvage pathway for NAD+) and NMNAT3 (Representative of key rate-constraining enzymes of de novo biosynthesis pathway for NAD+) in the process of NAD+ synthesis were compared in two groups. Protein levels of NAMPT in the G93A-Vehicle group were significantly lower than that in the WT-Vehicle group (Fig. 3B). However, reverse changes were observed in levels of NMNAT3 protein. NMNAT3 levels in brain cortex tissues of G93A-Vehicle group were significantly higher than that in the WT-Vehicle group (Fig. 3C). These above data implied that the NAD+ decline was related to susceptibility factors in the ALS, and suggested that the NAMPT-regulated NAD+ salvage synthesis pathway may be the primary explanation behind the NAD+ decline, while the protein of NMNAT that regulates the NAD+ denovo biosynthesis may increase as an adaptive response in the ALS.

### The NR treatment exhibited the better hanging wire endurance but did not delay the disease onset or extend the life span of ALS SOD1^G93A^ mice

**Figure 4:**
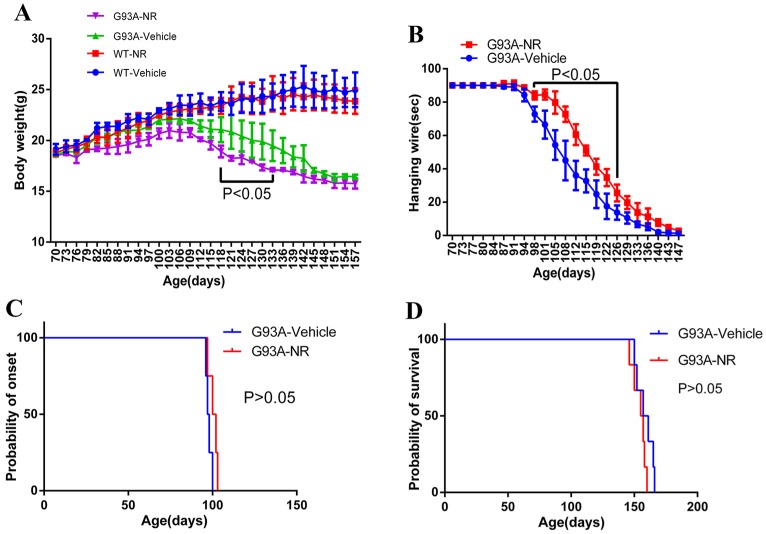
Effects of NR treatment on body weight, hanging wire, disease onset or life expectancy of ALS SOD1^G93A^ mice. **(A)** Body weight was different between G93A-NR and G93A-Vehicle groups treated from 50 days of age with significant values from 118 to 133 days of age. **(B)** Hanging wire was significantly different between G93A-NR and G93A-Vehicle groups from 98 to 126 days of age. **(C)** Disease onset (Defined by age at which hind limb tremors were apparent when suspending the mice in the air by its tail) in the G93A-NR group were not significantly different from G93A-Vehicle group mice. **(D)** G93A-NR mice showed no significant effect of survival time (The age when mice achieve the complete hind limb paralysis and have a failure to right itself once placed on its back) when compared with G93A-Vehicle mice. Data are expressed as mean ± SEM of n=4 mice/group; *P<0.05, **P<0.01, ***P<0.001, ****P< 0.0001.

Starting from the age of 70 days, mice were weighted the bodyweight every three days in successive 87 days. As a result, G93A-NR group mice showed significantly lower bodyweight than the G93A-Vehicle group from 118 days to 133 days (Fig. 4A). Motor functions were assessed using the hanging wire test. The G93A-NR treatment significantly exhibited better hanging wire endurance in the disease from 98 and 126 days of age when compared to G93A-Vehicle mice (Fig. 4B). G93A-NR mice treated with NR from 50 days of age had no significant extension in the disease onset when compared with litter-matched G93A-Vehicle mice (Fig. 4C). There was also no difference in the survival time between G93A-NR and G93A-Vehicle groups (Fig. 4D).

### The NR repletion promoted the clearance of mutant hSOD1 neurotoxic protein

NR is a naturally occurring precursor of NAD+ initially isolated from the fresh milk 49. The exogenous treatment with NR has been shown to increase intracellular NAD+ levels in an assortment of cell lines 50. Thus, we sought to determine in vivo the effect of NR administration on the hSOD1 aggregation in the brain cortex of SOD1^G93A^ ALS mice model. As expected, expression levels of hSOD1 protein were significantly reduced in the G93A-NR group compared with the G93A-Vehicle group (Fig. 1B). Interestingly, when compared protein levels of NAMPT and NMNAT3 between G93A-Vehicle and G93A-NR groups, we found that these were significantly increased the expression of NAMPT protein in the G93A-NR group (Fig. 3B). On the contrary, protein levels of NMNAT3 in the G93A-NR group were significantly reduced when compared with the G93A-Vehicle group (Fig. 3C).

### The NR treatment improved the proliferation, migration of NSCs/NPCs in the brain of ALS SOD1^G93A^ mice

Restoring the NAD+ content is not exclusively protecting neurons, since it also has been reported to prevent the stem cells exhaustion in vivo 51, 52. Data from several studies recommend that the NR treatment restores muscle, neuronal and melanocyte stem cell pools through the enlistment of UPR^mt^ and the synthesis of preclusion proteins, prompts the oxidative respiration and ATP levels and the higher mitochondrial layer potential 51-53. It also has been reported that the restoration of NAD+ in aged somatic cells improves the reprogramming proficiency and prolongs the life expectancy of mesenchymal stem cell by delaying senescence 53.

**Figure 5:**
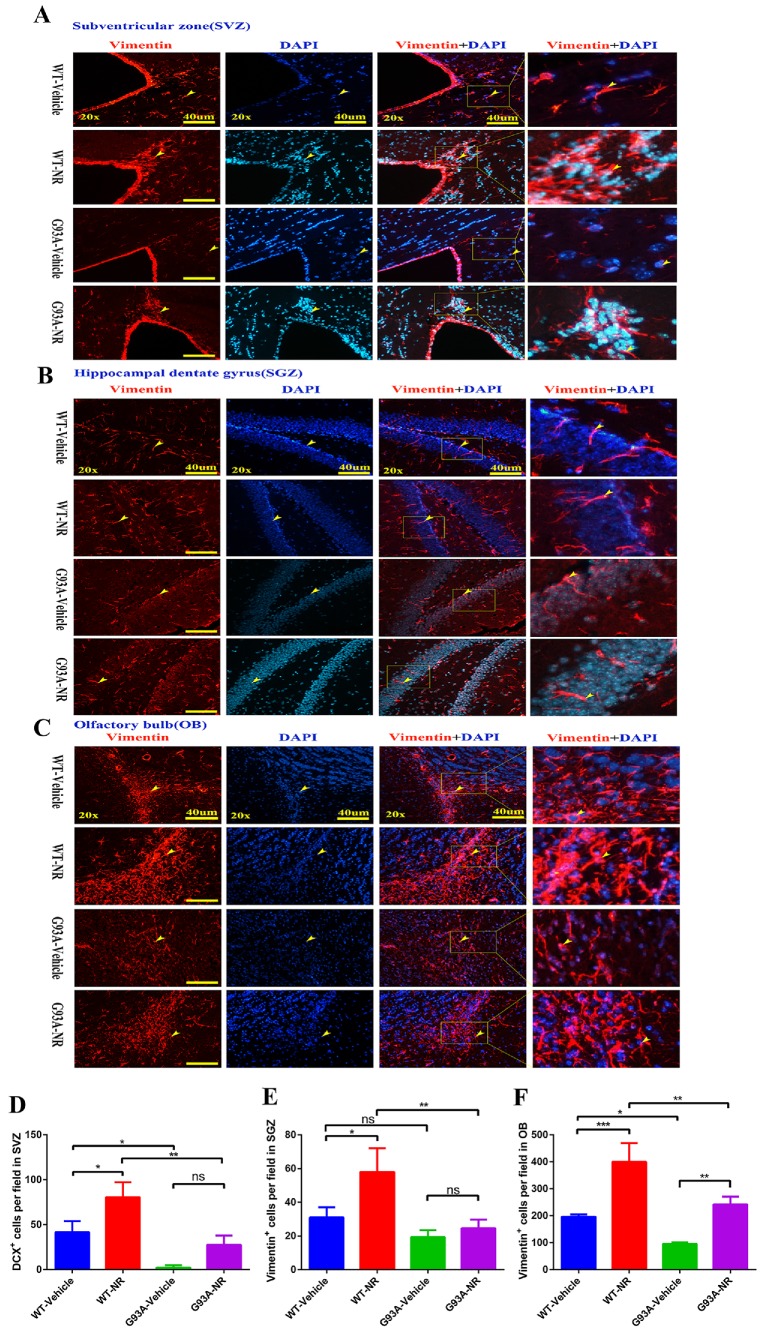
NR treatment increased the proliferation of NSCs/NPCs in the brain of ALS SOD1^G93A^ mice. Representative photomicrographs showed the immunostaining of vimentin (A cell proliferation marker; red) in the SVZ **(A)**, SGZ **(B)**, OB **(C)** at 120 days of four groups mice. Cell nuclei were counterstained with DAPI (blue). Yellow arrows indicated the colocalization of vimentin+ cell and DAPI. Bar graphs showed the analysis of vimentin+ cells in SVZ **(D)**, SGZ **(E)**, OB **(F)** regions. Scale bar: 40μm. Data were expressed as mean ± SEM of n=3 mice/group; *P<0.05, **P<0.01, ***P<0.001, **** P<0.0001.

Thus, we initially determined the impact of NR on the NSCs/NPCs proliferation in the SVZ, SGZ and OB of SOD1^G93A^ ALS mice brain at the age of 120 days. We performed the immunostaining of vimentin, a marker of NSCs/NPCs indicating neurogenesis. We found that the number of vimentin+ cells in the SVZ and OB, were significantly reduced in the G93A-Vehicle as compared with the WT-Vehicle group (Fig. 5D, F). And the number of vimentin+ cells in the SGZ of G93A-Vehicle had marginally reduction compared with WT-Vehicle mice (P=0.0518, Fig. 5E). Strikingly, the NR treatment in G93A-NR mice, significantly enhanced the number of vimentin+ proliferating cells in the SVZ, OB, as compared with G93A-Vehicle mice (Fig. 5D, F). However, the NR treatment to G93A-NR mice had no significantly effects on vimentin+ cells in the SGZ, when compared with G93A-Vehicle mice (Fig. 5E).

**Figure 6:**
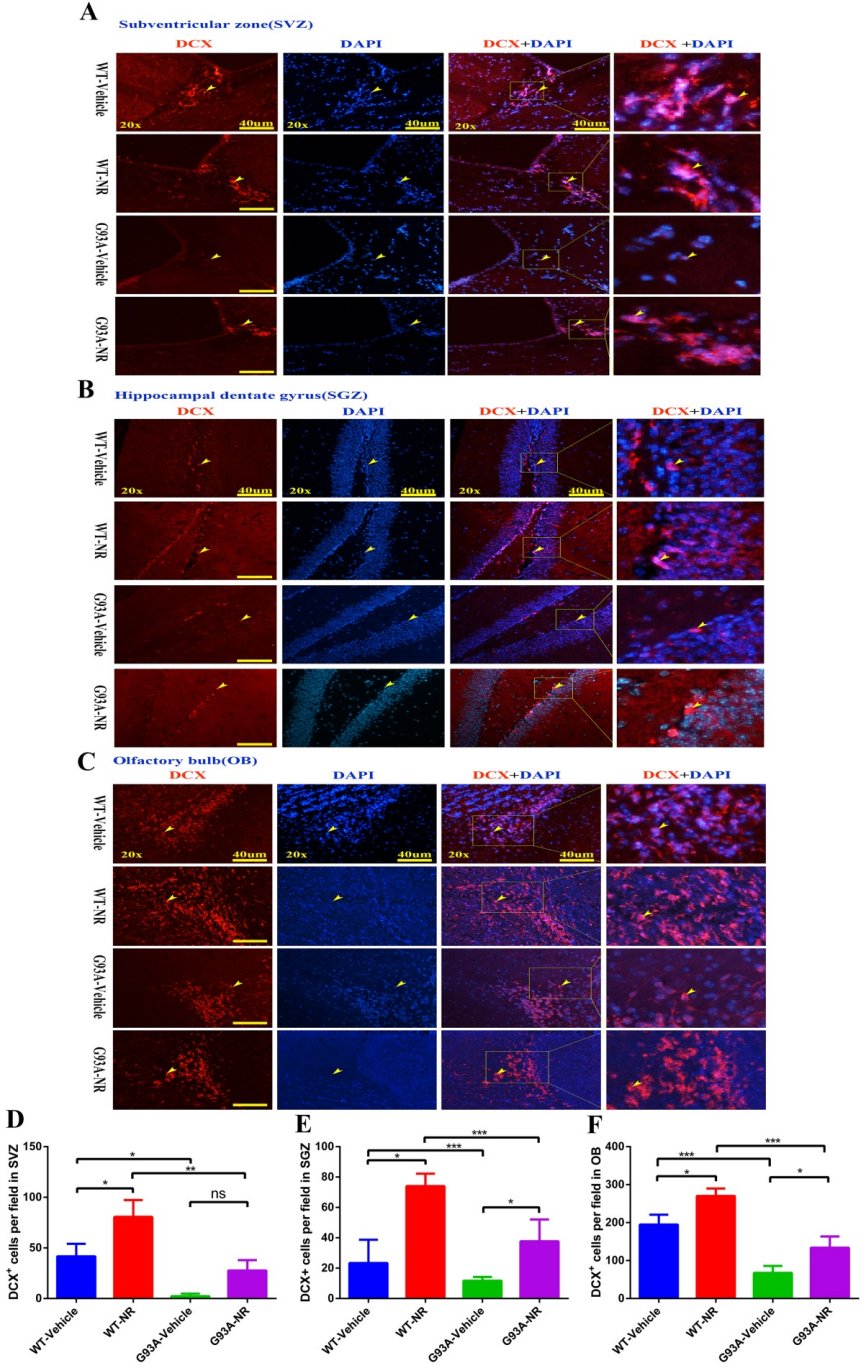
NR treatment extended the survival of newborn neurons in the brain of ALS SOD1^G93A^ mice. Representative photomicrographs showed the immunostaining of DCX (A maker of newborn neuron; red) and DAPI (blue) in SVZ **(A)**, SGZ **(B)**, OB **(C)** regions at 120 days from four groups mice. Cell nuclei were counterstained with DAPI. Yellow arrows indicated the colocalization of DCX+ cell and DAPI. Bar graphs showed the quantitative analysis of DCX+ cells in SVZ **(D)**, SGZ **(E)**, OB **(F)** regions. Scale bar: 40μm. Data were expressed as mean ± SEM of n=3 mice/group; *P<0.05, **P<0.01, ***P<0.001, ****P<0.0001.

Further, we performed the immunostaining of DCX, a marker of neuroblasts to inspect the impact of NR on immature neurons in ALS mice at the age of 120 days. Accordingly, the number of DCX+ cells was significantly reduced in the SVZ, OB of G93A-Vehicle group mice, as compared to the WT-Vehicle group (Fig. 6D, F). However, the number of DCX+ cells in the SGZ between G93A-Vehicle and WT-Vehicle mice had no statistical differences (Fig. 6E). Interestingly, the NR treatment to G93A-NR mice, significantly increased the number of DCX+ cells in the SVZ, SGZ and OB as compared to G93A-Vehicle mice (Fig. 6D, E, F). It is well definitely recommended that DCX+ cells migrate from SVZ to OB rostral migratory stream (RMS) means migration of immature neurons 54. Thus, the NR treatment tended to improve the migration of NPCs in the brain of SOD1^G93A^ mice.

To investigate whether NR mediated its effects via the mitochondrial UPR or the mitophagy pathway in the brain of SOD1^G93A^ ALS mice model, we investigated the effect of NR on LONP1, HSP60, and CLPP proteins, downstream target molecules of NAD+ metabolites. We found that levels of LONP1, HSP60 and CLPP proteins were significantly increased in the brain cortex of G93A-NR mice as compared to G93A-Vehicle mice (Fig. 2B, C, D). Nonetheless, we did not observe that mitophagy-related proteins (LC3-II, P62) have significantly differences between G93A-NR and G93A-Vehicle groups (Fig. 2F, G).

## Discussion

We obtained following several findings from our studied results: 1) The NR treatment exhibited the better hanging wire perseverance but did not postpone the disease onset or extended the life span of ALS SOD1^G93A^ mice. 2) The mutant hSOD1 protein mistakenly aggregated in the mitochondria of ALS SOD1^G93A^ mice cerebrum tissue, and the NR repletion promoted the clearance of mutant hSOD1 neurotoxic protein. 3) The mitochondrial function pathway was disrupted in the brain of ALS SOD1^G93A^ mice. 4) The inadequate function of NAD+ salvage synthesis pathway was the primary explanation behind the decline of NAD+ in the ALS SOD1^G93A^ mice. 5) The NR treatment enhanced the proliferation and migration of NSCs/NPCs in the brain of ALS SOD1^G93A^ mice. 6) Levels of UPR^mt^ related proteins were significantly increased in the brain of SOD1^G93A^ mice after the NR treatment.

Numerous molecular pathways have been ensnared in the neuronal death in ALS. Pathogenic mechanisms such as the oxidative stress, the glutamate excitotoxicity, the protein aggregation, the inflammation, apoptosis, the proteasomal and mitochondrial dysfunction happen during the disease progression 55-57. However, it is as yet unclear which of these occasions is the essential trigger for the beginning of ALS and which events occur as a result of initial activation of ALS disease. A substantial number of promising drugs had been preclinically found and indicated successful therapeutic effects in different ALS animal models, yet a large portion of them failed to demonstrate clinical viability. Two main approved drugs for the ALS disease treatment by FDA are riluzole and edaravone which just have marginal effects on clinic manifestations and the patient survival 28, 29, 58, 59. Accordingly, it is necessary to grow progressively effective and prudent methodologies for the ALS treatment.

The misfolded protein accumulation is a noteworthy pathological feature of ALS, which is distinguished in the brain and the spinal cord even at the presymptomatic stage, and may spread through the cell-to-cell transmission. The previous study proposed that NAD+ salvage pathway proteins could smother proteotoxicity in yeast models of neurodegeneration by advancing the clearance of misfolded proteins 35. In accordance with this research, our study showed the NR treatment reduced aggregated mutant hSOD1 proteins in the brain of SOD1^G93A^ mice, which indicted that NAD+ salvage pathway was a potential therapeutic target suppressing the collection of misfolded SOD1 in the ALS. More specifically, the mutant hSOD1 has been shown to associate with the mitochondria. It has been discovered that the mutant hSOD1 deposited on the cytoplasmic substance of spinal cord mitochondrial 45, accompanied by the modified mitochondria shape and the distribution in ALS 60. Similarly, our data demonstrated that obvious expression levels of hSOD1 protein in the mitochondria of SOD1^G93A^ mice, and the hSOD1 protein tended to be partly eliminated by the NR administration. Taken together, our observations provided evidences that the recharging of intracellular NAD+ by providing NR might decrease neurotoxic impacts of protein aggregates of mitochondria in the brain of ALS SOD1^G93A^ mice.

NAD+ is delivered in all eukaryotic cells. The basic intracellular NAD+ concentration can be up to 800 mM in yeast 61, 100-400 mM in human HEK293 cells 62, 63, and roughly 0.2 mmol/kg in the mouse tibialis foremost muscle 64. New strategies have been developed to empower the detection of subcellular NAD+ levels 62, 63, 65, 66. In our present study, we measured protein levels of NAMPT and NMNAT3 to indirectly assess the basal intracellular level of NAD+, for the reason they are key rate-limiting enzymes of salvage pathway and the de novo biosynthesis pathway for NAD+, respectively. It has been recently revealed that NAD+ levels decrease normally with age and involve in the support of mitochondrial health in the aged tissue 67, 68. Moreover, NAD+ levels decline with age in the hippocampus of mice, alongside the reduced expression of NAMPT 69. To examine whether the NAD+ decline was actually involved in the pathogenic mechanism of ALS disease, we measured expression levels of key proteins, NAMPT and NMNAT3 in SOD1^G93A^ mice and litter-matched WT mice, respectively. As a result, we observed protein levels of NAMPT in the cortical brain of SOD1^G93A^ mice were significantly reduced compared with WT mice. On the contrary, the expression level of NMNAT3 protein of cortical brain in the SOD1^G93A^ group was significantly upgraded compared with the WT group. Curiously, when compared protein levels of NAMPT and NMNAT3 between NR-treated and NR-untreated SOD1^G93A^ groups, we observed that these were significantly increased levels of NAMPT protein in G93A-NR mice compared with the G93A-Vehicle group. On the contrary, protein levels of NMNAT3 in the G93A-NR group were significantly decreased compared with G93A-Vehicle mice. In summary, our research confirmed that the NAD+ decline was related to susceptibility factors in the ALS, suggested that the insufficient NAMPT-mediated NAD+ salvage synthesis was the main reason for the NAD+ levels decline and the de novo biosynthesis of NAD+ might act as an adaptive response in the ALS.

Recent years have witnessed a resurgence of enthusiasm for the NAD+ biology. This has been driven to some degree by disclosing the intermediate of NAD+ biosynthesis, NR, effectively increases the NAD+ concentration in a variety of tissues, much of time with beneficial or therapeutic effects. The direct commitment of NR to the NAD+ metabolism was first perceived by Bieganowski and Brenner in 2004 70. This report described a class of enzymes known as NRKs (NR kinases) that could directly change over NR to NMN, bypassing the requirement for NAMPT in the salvage pathway. NR has additionally been reported to have a number of intriguing advantages in the CNS. One of studies to examine impacts of NR in vivo uncovered a striking improvement in the progression of Alzheimer's disease pathology in the Tg2576 model 71. NR also has been shown to prevent the noise-induced hearing loss and the neuritis withdrawal from hair cells in the internal ear through a SIRT3-dependent mechanism 72. NR additionally secures against the diabetic and chemotherapy-instigated neuropathy in mice 73, the raising expectations may have a role in the administration of chronic pain. In concordance with these outcomes, our data uncovered that the NR treatment displayed the better hanging wire endurance. One clarification for the better hanging wire test in the present investigation was that the morphological damage of muscle might be mitigated by the NR administration which was suggested by researches 20, 74, 75. Notably, it has been demonstrated that the NAD+/ sirtuin pathway adjusts the life span through the enactment of mitochondrial UPR and FOXO signaling 51. However, we observed no effects of NR administration on the lifespan in our study, suggesting the mechanism of longevity in the state of ALS disease may be different from that in the healthy. Besides, it is proposed that the maintenance of efficient DNA repair by NAD+ recharging in cells, c.elegans, and mice may delay the onset of aging and age-related diseases 76, 77. In contrary, our results indicated that the NR treatment did not postpone the disease onset of SOD1^G93A^ mice, which indicted that the difference might be the consequence of insufficient NAD+ supplementation or the course of treatment in our study.

There are two neurogenic niches in the mammalian adult brain: the SGZ of hippocampus and the SVZ, delivering new neurons that utilize the RMS to achieve OB. Cells in two discrete locales hold the ability to generate multiple lines composed of various cells, including NSCs, NPCs, newborn immature neurons and mature neurons. It has been recently reported that the NSCs and NPCs niche is altered in the ALS mice brain 32, 33, 78. Besides, the NR treatment has been shown to improve the stem cell function and somewhat relieve the muscle wasting phenotype in the mdx mice 20, 75. Furthermore, the NAD+ supplementation reestablished the mitochondrial function in NSCs during ageing and in the context of neurodegenerative disease 20, 22. Thus, we determined whether the NAD+ repletion with the NR supplementation have potential effects to promote the NSCs/NPCs proliferation or the immature neurons migration in the SVZ, SGZ and OB in SOD1^G93A^ mice. Intriguingly, in the present study, we observed that the NR treatment significantly attenuated the ALS-induced loss of NSCs/NPCs in SVZ, SGZ and OB. Meantime, the NR administration also continually renewed the pool of immature neurons, jointly suggesting the promotion to the proliferation and migration of NPCs/NSCs was improved by NR in the brain of SOD1^G93A^ mice. This may be clarified by that the NR treatment could reduce the stem cell exhaustion through the induction of UPR^mt^ in ALS SOD1^G93A^ mice.

## Conclusion

In summary, we conclude that the NR modulates the mitochondrial proteostasis and improves the adult neurogenesis through the activation of mitochondrial UPR signaling in the brain of SOD1^G93A^ mice, and NR may as a viable clinical therapeutic scheme of translational medicine for ALS and other neurodegenerative diseases. While many studies on NAD+ precursors are progressing, major issues remain. The pleotropic role of NAD+ in the human physiology is unpredictable and requires further mechanistic insight. For instance, a previous research proposed the NAD+ metabolism governs the proinflammatory senescence-related secretome and may animate the development of tumor cells 79. It is likewise conceivable that some unanticipated side effects may exhibit in certain human populations. Hence, profoundly stringent and carefully designed clinical trials are necessary to guarantee safety.

## Abbreviations

ALS: amyotrophic laterale sclerosis
NAD+: nicotinamide adenine dinucleotide
UPR^mt^: mitochondrial unfolded protein response
hSOD1: human SOD1
NSCs/NPCs: neural stem cells/neuronal precursor cells
NR: nicotinamide riboside
PH: Preiss-Handler
NAM: nicotinamide
NAMPT: nicotinamide monophosphoribosyl transferase
ATP: adenosine triphosphate
OXPHOS: oxidative phosphorylation system
SVZ: subventricular zone
SGZ: hippocampal dentate gyrus
OB: olfactory bulb
FDA: Food and Drug Administration
WT: wild-type
TG: transgenic
DCX: doublecortin
DTT: dithiothreitol
CNS: central nervous system
MQC: mitochondrial quality control
NMN: nicotinamide mononucleotide
SOD1: superoxide dismutase 1
OCT: optimum cutting temperature
SDS-PAGE: sulfate-polyacrylamide gel electrophoresis
PVDF: polyvinylidene fluoride
TBST: tris-buffered saline-Tween
RMS: rostral migratory stream
NRKs: NR kinases
