# Nicotinamide Riboside Enhances Mitochondrial Proteostasis and Adult Neurogenesis through Activation of Mitochondrial Unfolded Protein Response Signaling in the Brain of ALS SOD1^G93A^ Mice: Erratum

**DOI:** 10.7150/ijbs.71902

**Published:** 2022-03-03

**Authors:** Qi Zhou, Lei Zhu, Weiwen Qiu, Yue Liu, Fang Yang, Wenzhi Chen, Renshi Xu

**Affiliations:** 1 Department of Neurology, Jiangxi Provincial People's Hospital, Affiliated People's Hospital of Nanchang University, Nanchang 330006, Jiangxi, China; 2 Department of Neurology, First Affiliated Hospital of Nanchang University, Nanchang 330006, Jiangxi, China

In our paper, there were mistakes in Figure 5D and Figure 6D, E. They should be corrected as follows.

## Figures and Tables

**Figure 5:**
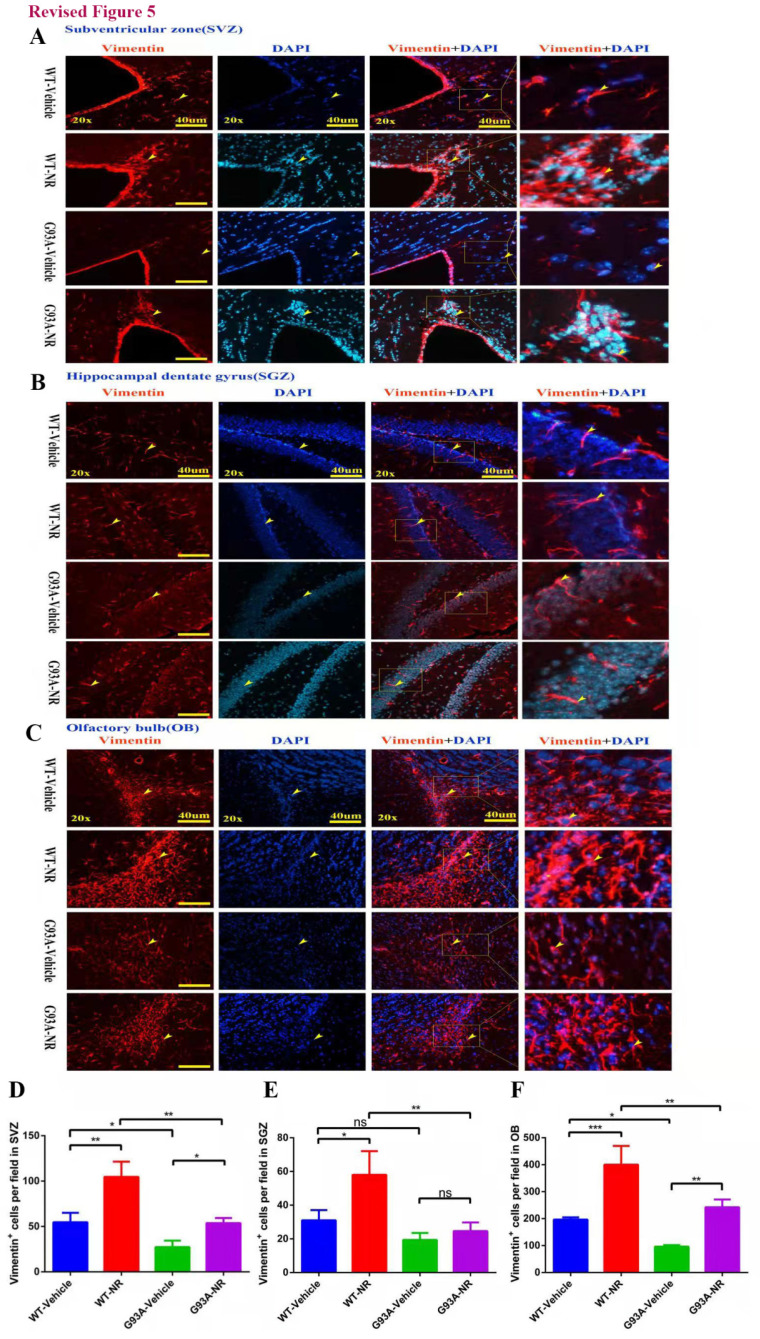
NR treatment increased the proliferation of NSCs/NPCs in the brain of ALS SOD1G93A mice. Representative photomicrographs showed the immunostaining of vimentin (A cell proliferation marker; red) in the SVZ **(A)**, SGZ **(B)**, OB **(C)** at 120 days of four groups mice. Cell nuclei were counterstained with DAPI (blue). Yellow arrows indicated the colocalization of vimentin+ cell and DAPI. Bar graphs showed the analysis of vimentin+ cells in SVZ **(D)**, SGZ **(E)**, OB **(F)** regions. Scale bar: 40μm. Data were expressed as mean ± SEM of n=3 mice/group; *P<0.05, **P<0.01, ***P<0.001, **** P<0.0001.

**Figure 6:**
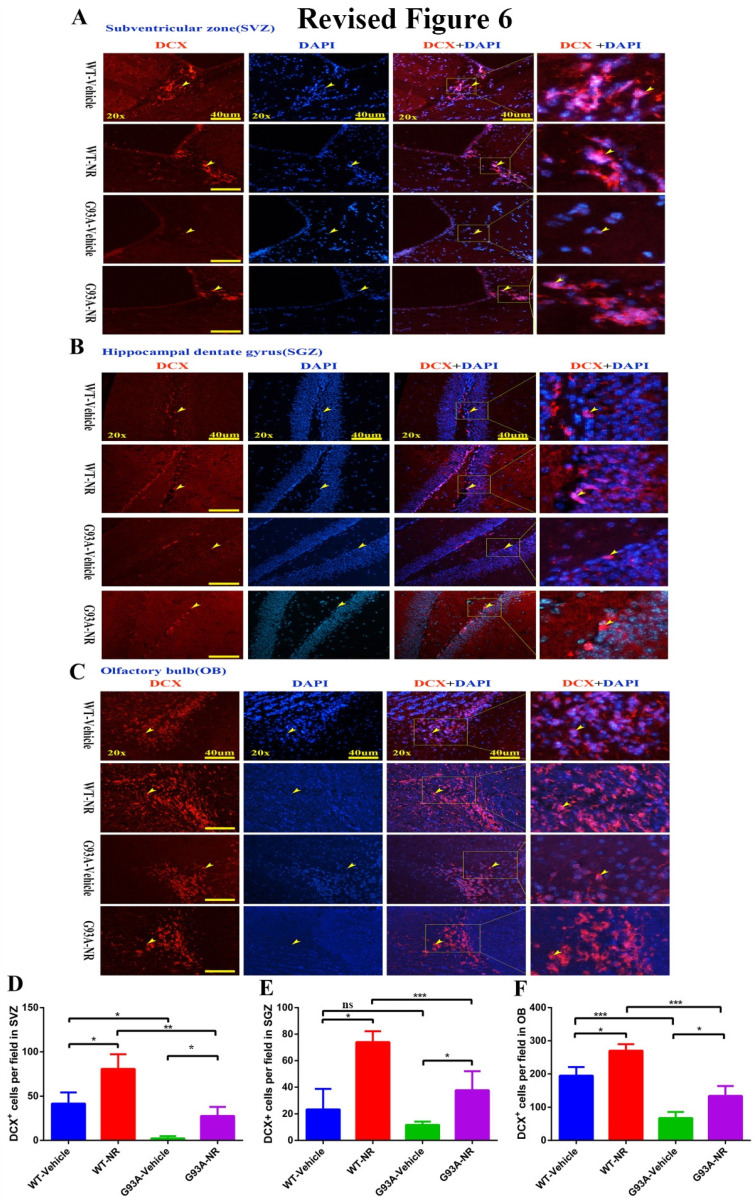
NR treatment extended the survival of newborn neurons in the brain of ALS SOD1G93A mice. Representative photomicrographs showed the immunostaining of DCX (A maker of newborn neuron; red) and DAPI (blue) in SVZ **(A)**, SGZ **(B)**, OB **(C)** regions at 120 days from four groups mice. Cell nuclei were counterstained with DAPI. Yellow arrows indicated the colocalization of DCX+ cell and DAPI. Bar graphs showed the quantitative analysis of DCX+ cells in SVZ **(D)**, SGZ **(E)**, OB **(F)** regions. Scale bar: 40μm. Data were expressed as mean ± SEM of n=3 mice/group; *P<0.05, **P<0.01, ***P<0.001, ****P<0.0001.

